# Placental Bioenergetics and Antioxidant Homeostasis in Maternal Obesity and Gestational Diabetes

**DOI:** 10.3390/antiox13070858

**Published:** 2024-07-18

**Authors:** Chiara Mandò, Sara Castiglioni, Chiara Novielli, Gaia Maria Anelli, Anaïs Serati, Francesca Parisi, Chiara Lubrano, Monica Zocchi, Roberta Ottria, Matteo Giovarelli

**Affiliations:** 1 Department of Biomedical and Clinical Sciences, Università degli Studi di Milano, 20157 Milan, Italy; chiara.mando@unimi.it (C.M.); sara.castiglioni@unimi.it (S.C.); gaia.anelli@unimi.it (G.M.A.); francesca.parisi@unimi.it (F.P.); chiara.lubrano@unimi.it (C.L.); monica.zocchi@unimi.it (M.Z.); roberta.ottria@unimi.it (R.O.); matteo.giovarelli@unimi.it (M.G.); 2 Department of Woman, Mother and Neonate, Buzzi Children’s Hospital, ASST Fatebenefratelli Sacco, 20154 Milan, Italy

**Keywords:** obesity, gestational diabetes, placenta, oxidative status, bioenergetics, mitochondria

## Abstract

Maternal obesity has been associated with short- and long-term risks of pregnancy-perinatal adverse events, possibly due to alterations of placental mitochondrial bioenergetics. However, several detrimental mechanisms occurring in the placentas of women with obesity still need to be clarified. Here, we analyzed placental mitochondrial features and oxidative environment of 46 pregnancies in relation to pre-pregnancy BMI. Seventeen Caucasian normal-weight (NW) and twenty-nine women who were obese (OB) were enrolled. The protein expression of mitochondrial CypD and electron transfer chain complexes (C) I–V were measured, as well as ATP production and oxygen consumption rates (OCRs). The protein levels of the pro/anti-oxidant enzymes TXNIP, SOD2, and PON2 were also analyzed. Despite no differences in CypD expression, OCRs were significantly lower in OB vs. NW women. Accordingly, ATP synthase (CV) levels and ATP content were decreased in OB women, positively correlating with placental efficiency, suggesting a link between ATP deficiency and placental dysfunction. SOD2 expression negatively correlated with maternal BMI, indicating a possible impairment of antioxidant defenses with increasing BMI. These changes were worsened in 10 OB women presenting with gestational diabetes mellitus. Overall, these results suggest alterations of placental bioenergetics in pregnancies of women with obesity, possibly leading to placental dysfunction and altered fetal development and programming.

## 1. Introduction

The 2023 World Obesity Atlas [1] showed alarming rates of overweight and obesity worldwide, suggesting that over 4 billion people may be affected by 2035, compared with over 2.6 billion in 2020. This leads to increased risks of non-communicable disease and a consequent global economic impact.

Becoming pregnant with obesity involves numerous risks, with short- and long-term consequences both for the mother and the baby. Many studies reported associations between maternal obesity and increased risks of pregnancy-perinatal adverse events, with lifelong consequences for the offspring [2,3,4,5,6,7,8,9]. Indeed, the fetal programming can be deranged by the maternal obese environment [10,11,12,13,14,15]. Obesity acts on many cellular mechanisms, playing a pivotal role in the pathogenesis of inflammaging [12,16] and establishing a vicious negative circle characterized by systemic oxidative stress and chronic low-grade inflammation. During pregnancy, the physiological increase in inflammatory cytokines and insulin resistance is exacerbated in the presence of maternal obesity [17,18,19,20]. These conditions heighten the risk of adverse pregnancy outcomes, most notably gestational diabetes mellitus (GDM) [21,22,23,24], also representing a key mechanism involved in Type 2 Diabetes Mellitus occurrence and cardiovascular diseases after pregnancy [25].

The placenta is a high energy-demanding and metabolically active tissue as it allows oxygen and nutrient supply to the fetus and waste removal. The placental efficiency can be affected by maternal obesity, leading to altered fetal development and programming [26,27,28,29,30,31,32,33,34,35]. However, many of the molecular mechanisms occurring in the placentas of women with obesity still need to be clarified. Previous studies reported alterations of the placental mitochondrial bioenergetics and oxidative status in pregnancies with placental insufficiency [36,37,38,39,40]. Reactive oxygen species (ROS) are by-products of oxidative phosphorylation (OXPHOS), which takes place in the mitochondrial inner membrane. OXPHOS occurs via the electron transport chain, which is composed of five multi-subunit protein complexes responsible for the coupled electron and proton transfer across the membrane, powering oxygen consumption and ATP synthesis [41]. Alterations in OXPHOS capacity may, therefore, lead to excessive ROS production and oxidative stress, which in turn can impair mitochondrial function and ATP generation. Indeed, only a small fraction of ATP is produced by cytoplasmic glycolysis, whereas the majority of ATP production occurs at the level of the mitochondria.

The aim of this study was to elucidate some of the placental alterations occurring in women with pregestational obesity, with a specific focus on bioenergetics and the oxidative environment. Indeed, placental mitochondria play a key role in maintaining pregnancy [37,38,42]. Identifying specific impairments of the placental bioenergetic and oxidative state in pregnancies of women with obesity could, therefore, represent an important therapeutic target for fetal reprogramming.

## 2. Materials and Methods

### 2.1. Population

Pregnant women were enrolled in the Department of Woman, Mother and Child of the Vittore Buzzi Children’s Hospital (ASST Fatebenefratelli-Sacco) in Milan.

The study was conducted in accordance with the Declaration of Helsinki and in compliance with all current Good Clinical Practice guidelines, local laws, regulations, and organizations. The protocol was approved by the Hospital ethical committee (Prot. N. 17739/2018). All participants provided their informed consent to collect personal data and biological samples.

Forty-six Caucasian pregnant women with spontaneous single-term pregnancies were consecutively recruited at the time of elective cesarean section. Gestational age was calculated from the last menstrual period and confirmed by an ultrasound crown-rump length measurement performed at 11–13 gestational weeks [43].

The enrolled patients were grouped in relation to pregestational Body Mass Index (BMI, kg/m^2^), according to the 2009 Institute of Medicine (IOM) guidelines [44]: healthy Normal-Weight (NW) women (18.5 ≤ BMI < 25, n = 17) and Obese (OB) women (BMI ≥ 30, n = 29).

Gestational diabetes mellitus (GDM) was diagnosed in a subgroup of OB women (OB GDM(+), n = 10) according to the International Federation of Gynecology and Obstetrics (FIGO) guidelines, by the Oral Glucose Tolerance Test (OGTT-75 g) performed at 24–28 weeks of gestation [45,46]. Briefly, the OGTT procedure consisted of a fasting blood glucose test (OGTT I) and then glycemia checks after 60 min (OGTT II) and after 120 min (OGTT III) from the administration of 75 g of anhydrous glucose dissolved in 300 mL of water. Blood glucose quantification was performed in maternal venous blood using a standardized clinical biochemistry laboratory dosage using enzymatic spectrophotometric analysis (the hexokinase/glucose-6-phosphate dehydrogenase (g6pd) method). GDM was diagnosed for one or more glycemic curve values higher than 92,180,153 mg/dL, respectively, at 0, 60, and 120 min. Only diet-controlled GDM without medication was included in the present study.

Any maternal chronic comorbidity (i.e., chronic hypertension, autoimmune diseases, or pregestational diabetes) or pregnancy complications different from GDM (e.g., preeclampsia, infections, or congenital/genetic abnormalities), non-Caucasian ethnicity, maternal smoking/alcohol use and ART conception represented exclusion criteria. Women undergoing any pharmacological therapy in pregnancy were additionally excluded, including metformin and insulin.

Maternal and neonatal clinical characteristics were recorded.

Maternal and cord blood values were measured in a subgroup of pregnancies (13 NW and 21 OB), as previously described [47]. Briefly, both umbilical venous blood (UV, which transports oxygen and nutrients from the maternal to the fetal compartment) and umbilical arterial blood (UA, blood flow from the fetus towards the mother) were immediately sampled from a doubly clamped segment of the cord. All samples were collected in heparinized syringes that were sealed and stored on ice. Blood gases (pO_2_ and pCO_2_), pH, O_2_ saturation, lactate, and glucose concentrations were measured using a blood/gas/electrolyte analyzer.

### 2.2. Placental Tissue Collection

Placentas were collected in sterile conditions immediately after cesarean section, cleaned of excess blood, and weighed after the membranes and cord were discarded from the disc. Biometric measurements were performed as previously described [48]. Briefly, the placental area was estimated by calculating the area of an ellipse from the diameters (D × d × *π*/4). Assuming constant density, the placental thickness was obtained as the weight divided by area. Placental efficiency was calculated as the ratio between neonatal weight and placental weight.

Chorionic villi biopsies were sampled from different sites of the placental disc (central, median, and peripheral) on the maternal side. The maternal decidua was carefully discarded from the maternal side of the placenta, and the villous portion of the tissue was picked up by coring 1 cm^3^ biopsies. Samples were thoroughly washed with phosphate-buffered saline to eliminate excessive blood and either immediately stored at −80 °C for protein expression and ATP production analysis or placed in ice-cold BIOPS buffer for cryopreservation and subsequent analysis by high-resolution respirometry (HRR) [49].

### 2.3. Protein Expression Analysis—SDS PAGE and Western Blot

Proteins were extracted from placental biopsies of 16 NW, 18 OB GDM(−), and 10 OB GDM(+) women.

Total protein extracts were obtained with a lysis buffer (50 mM Tris–HCl pH 7.4, 150 mM NaCl, 1% NP-40, 0.25% Na-deoxycholate) with added protease inhibitors (10 μg/mL of Leupeptin, 10 μg/mL of Aprotinin and 1 mM of Phenylmethylsulfonyl fluoride, PMSF) and phosphatase inhibitors (1 mM of sodium fluoride, 1 mM of sodium vanadate, 5 mM of sodium phosphate). A syringe was used to obtain better homogenization of the samples. Total protein extracts were quantified with a Bradford assay and 20–30 μg of proteins were separated using an SDS-PAGE on Mini-PROTEAN TGX Stain-free Gels (Bio-Rad, Hercules, CA, USA) and transferred to nitrocellulose membranes using a Trans-Blot TurboTM Transfer Pack (Bio-Rad). The stain-free membrane was acquired with a ChemiDoc MP Imaging System (Bio-Rad) and used as a loading control.

After blocking with bovine serum albumin (BSA), western blot analysis was performed using primary antibodies against the antioxidant enzymes Paraoxonase 2 (PON2) and Superoxide Dismutase 2 (SOD2), the pro-oxidant thioredoxin interacting protein (TXNIP), the mitochondrial protein cyclophilin D (CypD), and actin. Details on primary antibodies (product number and manufacturer, dilution) are reported in Supplementary Table S1. Mitochondrial electron transfer system complexes I–IV and ATP synthase (complex V) expression were analyzed with oxPhos Rodent WB antibody cocktail (45-8099, Thermo Fisher Scientific, Waltham, MA, USA).

After extensive washing, nitrocellulose membranes were incubated with secondary antibodies conjugated with horseradish peroxidase (GE Healthcare, Waukesha, WI, USA). The immunoreactive proteins were detected using a Clarity™ Western ECL substrate (Bio-Rad), and images were acquired using a ChemiDoc MP Imaging System (Bio-Rad). Densitometry of the bands was performed using the software ImageLab (version 6.0.1 build 64 standard edition, Bio-Rad).

### 2.4. Placental ATP Production

A luminescence assay (CellTiter-Glo Luminescent Cell Viability Assay, G7570, Promega, Madison, WI, USA) was used to determine the ATP content in placental specimens [13 NW, 14 OB GDM(−) and 10 OB GDM(+)]. Specifically, frozen placentas (~20 mg) were homogenized in 0.3 mL of cold lysis buffer (0.25 M sucrose, 10mM of HEPES-NaOH at pH 7.4), with ultra-turrax (10 s at max speed), and the homogenates were cleared by centrifugation at 1000× *g*, at 4 °C for 10 min. A total of 250 µL of supernatant was quickly added to an equal volume of ice-cold 10% trichloroacetic acid (TCA), shaken for 20 s, and then centrifuged for 10 min at 10,000× *g* at 4 °C. After TCA extraction, TCA was neutralized adding 200 μL of Tris-acetate buffer (1 M pH 8) to 400 µL of supernatant. Following a 10-fold dilution with deionized water, the extract was used for luciferin-luciferase assay. The reaction mix, containing luciferase and substrate, was added, and the light emission was measured using a GloMax luminometer (Promega) and quantified according to an ATP standard curve. ATP values (nM) were normalized on protein content [50,51,52].

### 2.5. Mitochondrial Respiration Analysis—High-Resolution Respirometry (HRR) 

Oxygen Consumption Rates (OCRs) of placental specimens [5 NW and 6 OB GDM(−)] were measured in the 2 mL O2K oxygraph chambers (Instruments Oroboros, Innsbruck, Austria) at 37 °C in a stirring MIR06 buffer [49,53]. The HRRs were performed using ~20 mg wet weight of placental tissue with a high oxygen concentration (400 μM) setting by the addition of hydrogen peroxide. OCRs were expressed in pmol O_2_/s·mg of sample to measure the steady-state oxygen fluxes (respiratory rates). A specific Substrate-Uncoupler-Inhibitor-Titration (SUIT) protocol 11, with a few modifications, was used. Pyruvate (5 mM), glutamate (10 mM), and malate (2 mM) were added to determine LEAK respiration (L). Then, the complex I (CI) oxidative phosphorylation (OXPHOS) capacity was stimulated by the addition of ADP (2.5 mM). The addition of cytochrome C (10 µM) was performed to test the integrity of the outer mitochondrial membrane. Subsequent titration with succinate (10 mM) was used to evaluate the maximal oxidative phosphorylation capacity through complexes I and II (CI + II). The maximal capacity of the Electron Transfer System (ETS) was evaluated by 0.5 μM steps titration of the uncoupler protonophore carbonyl cyanide p-trifluoro-methoxyphenyl hydrazone (FCCP). Uncoupled complex II-linked respiration (CII) was achieved by the addition of the CI inhibitor rotenone (0.5 µM). The respiratory system was inhibited with the complex III inhibitor antimycin A (2.5 µM) to obtain the non-mitochondrial residual oxygen consumption flux (ROX). Complex IV (CIV) activity was stimulated using N,N,N0,N0-Tetramethyl-p-phenyl-enediamine dihydrochloride (TMPD) (0.5 μM) and ascorbate (2 mM), recorded for 5 min, and hence stopped with the addition of CIV inhibitor sodium azide (100 mM) to calculate the TMPD autoxidation oxygen consumption. Oxygen fluxes were corrected by subtracting ROX from each steady state. The DatLab7 software (version 7, Instruments Oroboros, Innsbruck, Austria) was used for data acquisition and analysis.

### 2.6. Statistical Analysis

The database was cleaned by eliminating the outliers after double-checking with the original anamnestic questionnaire and correcting typing errors.

Maternal characteristics, placental and fetal/neonatal data, protein expression data, and ATP levels were compared among groups using a One-way ANOVA/Student’s *t*-test or the Kruskal–Wallis/Mann–Whitney U test according to data distribution (assessed by the Kolmogorov–Smirnov test). In post-hoc analyses, Tukey’s HSD test and the Mann–Whitney U test with Bonferroni correction (thus considering statistical significance when *p* ≤ 0.017) were used.

Grouped analyses of mitochondrial OCR states were performed using a two-way ANOVA followed by post-hoc Tukey’s multiple comparisons test.

The results are expressed as the means ± standard deviation of the indicated values.

Correlations between variables were assessed using the Spearman rank order correlation. Differences and correlations were considered significant when *p* < 0.05.

Analyses were performed using the statistical package SPSS, v.29 (IBM; Armonk, NY, USA). High-resolution Respirometry results were analyzed using GraphPad Prism software package (version 8.4, Graph Software, La Jolla, CA, USA).

## 3. Results

### 3.1. Clinical Data

Clinical characteristics of the study population are summarized in Table 1 and Table 2.

The included groups were homogeneous for maternal age and parity. All pregnancies delivered at term, between 38 + 2 and 40 weeks of gestation, as an inclusion criterion.

OB women gained significantly less weight compared to NW women (*p* < 0.01), as recommended by the Institute of Medicine [44].

However, OB women had significantly heavier placentas than NW women (*p* < 0.05).

**Figure 1:**
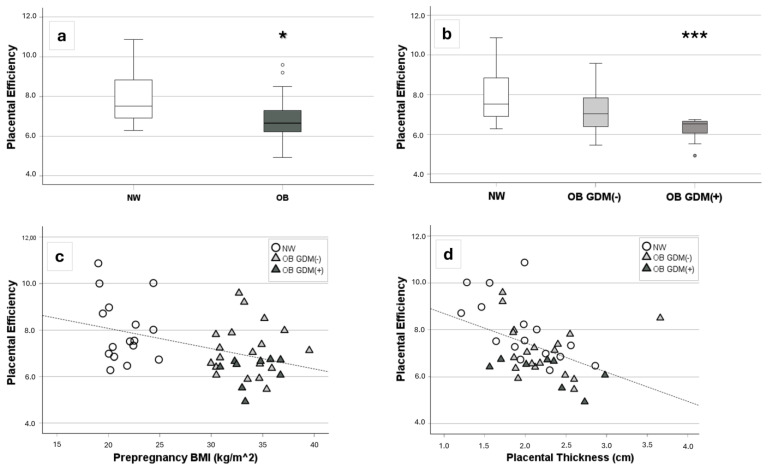
Placental efficiency in the study population [17 NW, 19 OB GDM(−), 10 OB GDM(+)]. (**a**,**b**) Comparison of placental efficiency, calculated as neonatal/placental weight ratio, among (**a**) OB and NW, and (**b**) OB GDM(+), OB GDM(−), and NW groups. Data are shown as box plots, indicating the median and the 25th and 75th percentiles. Placental efficiency resulted significantly lower in OB ((**a**), * *p* < 0.05 vs. NW—Mann–Whitney U test), and specifically in OB GDM(+) ((**b**), *** *p* < 0.001 Mann–Whitney U test as post-hoc analysis following Kruskal–Wallis test), compared to NW. (**c**,**d**) Significant correlations between placental efficiency and (**c**) maternal pre-pregnancy BMI (r = −0.38, *p* = 0.011) or (**d**) placental thickness (r = −0.52, *p* < 0.001) (Spearman rank order correlation).

Furthermore, placental efficiency was significantly lower in OB women compared to NW women (*p* < 0.05) (Figure 1a), with women affected by GDM having increasingly less efficient placentas vs. NW women (*p* < 0.001) (Figure 1b). Moreover, placental efficiency was significantly inversely related to pre-pregnancy BMI (r = −0.38, *p* = 0.01), as well as to placental thickness (r = −0.52, *p* < 0.001) (Figure 1c,d).

While umbilical artery (UA) and umbilical vein (UV) pH were not different among the analyzed groups, UV pO_2_ values were significantly lower (*p* = 0.045) and UV pCO_2_ significantly higher (*p* = 0.047) in OB vs. NW women. UV O_2_ saturation also tended to be lower in OB women, and UV lactate was slightly higher in OB vs. NW women, though not reaching statistical significance.

OGTT-II and III values were higher in OB GDM(+) women compared to healthy women, as expected.

### 3.2. Molecular Data

Detailed protein expression data values are reported in Supplementary Table S2.

#### 3.2.1. Cyclophilin D Protein Expression

**Figure 2:**
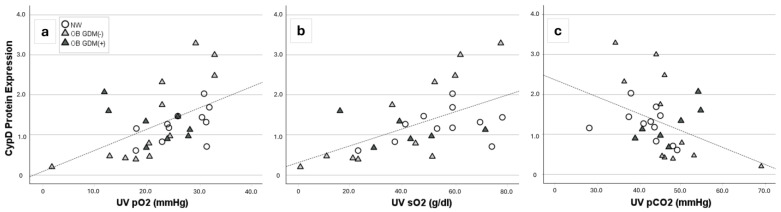
CypD protein expression in placenta of 16 NW, 18 OB GDM(−), 10 OB GDM(+). Significant correlations between CypD placental expression and umbilical vein (**a**) pO_2_ (r = +0.51, *p* = 0.004), (**b**) O_2_ saturation (r = +0.56, *p* = 0.002) or (**c**) pCO_2_ (r = −0.43, *p* = 0.018) (Spearman rank order correlation).

CypD placental protein expression was not different among groups. Nevertheless, it was significantly positively related to both UV pO_2_ (r = 0.51, *p* = 0.004) and UV satO_2_ (r = 0.56, *p* = 0.002) and negatively correlated with UV pCO_2_ (r = −0.43, *p* = 0.018) (Figure 2).

#### 3.2.2. Mitochondrial Respiratory Chain Proteins Expression

**Figure 3:**
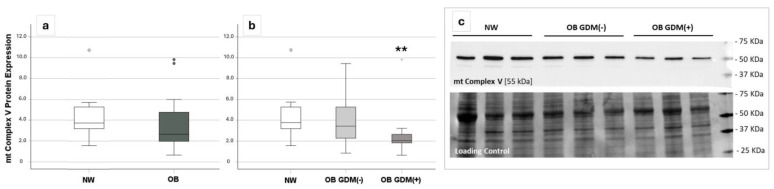
Mitochondrial complex V (mt-C-V) protein expression in the placenta of *16 NW, 18 OB GDM(−), 10 OB GDM(+)*. Comparison of placental mt-C-V expression among (**a**) OB and NW (Mann–Whitney U test), (**b**) OB GDM(+), OB GDM(−), and NW groups. Data are shown as box plots, indicating the median and the 25th and 75th percentiles. mt-C-V expression resulted significantly lower in OB GDM(+) placentas ((**b**), ** *p* < 0.01 vs. NW—Mann–Whitney U test as post-hoc analysis following Kruskal–Wallis test). (**c**) Western blot of three representative samples for each group of mt-C-V, and stain-free membrane as loading control.

The expression of placental mitochondrial complex V (mt-C-V), the downstream protein complex directly involved in ATP production, tended to be lower in OB vs. NW women, though it did not reach statistical significance. However, when considering OB, depending on the presence of GDM, mt-C-V expression was significantly different across groups (*p* = 0.05), with decreasing values in the presence of GDM. The post-hoc test showed significantly lower levels in OB GDM(+) compared to NW women (*p* = 0.008) (Figure 3).

Placental complex V expression was also positively correlated with the umbilical vein glucose concentration (r = 0.46, *p* = 0.009).

No significant differences were found in the expression of placental mitochondrial complexes I, II, III, and IV. 

#### 3.2.3. Pro/Antioxidant Proteins Expression

SOD2, PON2, and TXNIP proteins, the first two involved in the anti-oxidant defense of the cells and the latter a pro-oxidant triggering ROS generation, did not show any significant difference in expression.

However, SOD2 expression was significantly negatively correlated with maternal pre-pregnancy BMI (r = −0.41; *p* = 0.006).

TXNIP levels were significantly positively correlated with the placental thickness and negatively correlated with the placental surface (r = +0.37, *p* = 0.01; r = −0.38, *p* = 0.01, respectively).

#### 3.2.4. ATP Production

Placental ATP levels were measured in 13 NW and 24 OB women [14 OB GDM(−) and 10 OB GDM(+)].

**Figure 4:**
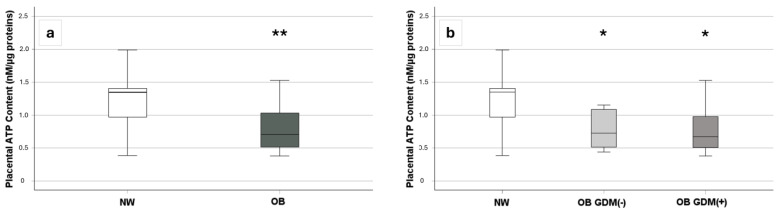
Placental ATP levels across groups [13 NW, 14 OB GDM(−), 10 OB GDM(+)]. Comparison of placental ATP levels among (**a**) OB and NW, and (**b**) OB GDM(+), OB GDM(−), and NW groups. Data are shown as box plots, indicating the median and the 25th and 75th percentiles. ATP levels resulted significantly lower in OB placentas ((**a**), ** *p* < 0.01 vs. NW—Mann–Whitney U test)—and, in more details (**b**), both in OB GDM(−) and in OB GDM(+) compared to NW (* *p* < 0.05 Mann–Whitney U test as post-hoc analysis following Kruskal–Wallis test).

In accordance with the trend of complex V protein levels (Figure 3), ATP levels were significantly lower in OB compared to NW placentas (*p* = 0.010) (Figure 4a). When analyzing data considering OB groups depending on the presence or the absence of GDM, the Kruskal–Wallis test showed a statistically significant difference in placental ATP levels across groups (*p* = 0.04). The post-hoc analysis revealed that NW had significantly higher levels compared to both OB GDM(−) (*p* = 0.02) and OB GDM(+) groups (*p* = 0.04) (Figure 4b).

**Figure 5:**
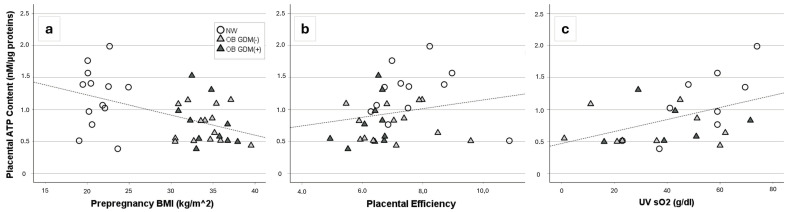
Placental ATP levels. Significant correlations between placental ATP levels and (**a**) maternal pre-pregnancy BMI (r = −0.43, *p* = 0.009), (**b**) placental efficiency (r = +0.41, *p* = 0.017), or (**c**) umbilical vein O_2_ saturation (r = +0.41, *p* = 0.047) (Spearman rank order correlation).

Placental ATP was significantly negatively correlated with pre-pregnancy BMI (r = −0.43, *p* = 0.009) and positively correlated with placental efficiency (r = +0.41, *p* = 0.017) and with the umbilical vein oxygen saturation (r = +0.41, *p* = 0.047) (Figure 5).

#### 3.2.5. Mitochondrial Respiration

**Figure 6:**
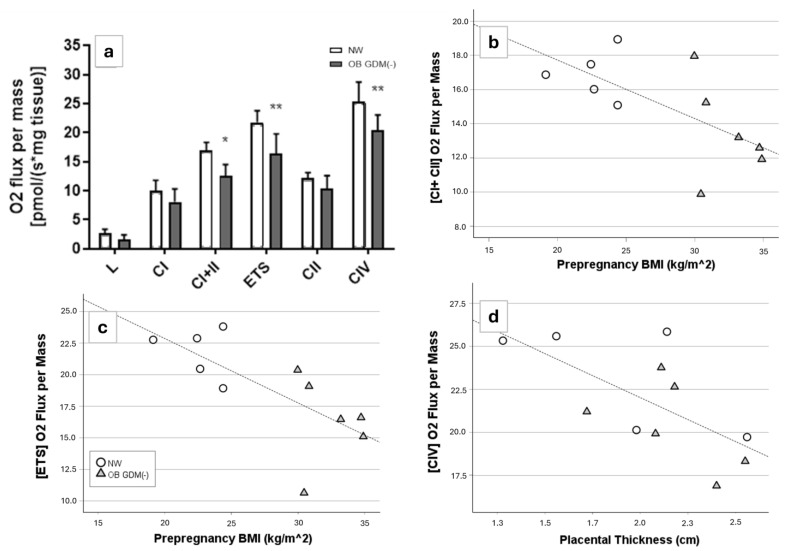
Placental oxygen consumption rate values [5 NW, 6 OB GDM(−)]. Oxygen flux values (pmol/s·mg tissue) of NW and OB GDM(−) placentas in the indicated respiratory states (L: leak state; CI: complex I activity; CI + II: complex I + II activity; ETS: electron transfer system maximal capacity; CII: complex II activity; CIV: complex IV activity). (**a**) Values are expressed as mean ± standard deviation; * *p* < 0.05, ** *p* < 0.01 vs. NW placentas (post-hoc Tukey’s multiple comparisons test following ANOVA). (**b**,**c**) Significant correlations between maternal pre-pregnancy BMI and O_2_ flux values of placentas in (**b**) complex I + II activity (r = −0.67, *p* = 0.025) or (**c**) electron transfer system maximal capacity (r = −0.72, *p* = 0.013). (**d**) Significant correlation between placental thickness and O_2_ flux values in complex IV activity (r = −0.67, *p* = 0.025). Correlations between variables were assessed using the Spearman rank order correlation.

Respirometric analysis was performed in NW and OB GDM(−) placentas, indicating a reduced maximal oxygen consumption rate in OB GDM(−) placentas compared to NW. In particular, the ATP production-coupled OXPHOS capacity, sustained by complexes I and II, was significantly lower in OB GDM(−) placentas (*p* < 0.05) as well as the uncoupled mitochondrial maximal capacity state (ETS) (*p* < 0.01). Moreover, complex IV activity was impaired in OB GDM(−) placentas (*p* < 0.01) globally, highlighting a diffuse defect throughout the mitochondrial electron transport system (Figure 6a).

The measured oxygen consumption values of the complexes positively correlated with each other (*p* < 0.01).

Values measured for CI + CII and ETS were significantly inversely correlated with maternal pre-pregnancy BMI (r = −0.67, *p* = 0.025 and r = −0.72, *p* = 0.013, respectively), while complex IV activity negatively correlated with the placental thickness (r = −0.67, *p* = 0.025) (Figure 6b–d).

## 4. Discussion

Although women with obesity and GDM differ in some clinical and metabolic characteristics from women with obesity without GDM, they share a common obesity-driven altered metabolic profile characterized by increased inflammatory cytokines, insulin resistance, fatty acids, and further alterations, compared to healthy pregnant women with a normal weight [17,18,54]. These conditions may affect placental bioenergetics and oxidative status in all women with obesity, with increasing severity depending on the further coexistence of gestational diabetes mellitus [18,54,55]. Therefore, we first performed analyses by comparing all pregnant women with obesity to normal-weight controls, and we then tested whether the increased metabolic severity in GDM could differently impact on the analyzed parameters.

Many of the mechanisms underlying the association between maternal obesity and intrauterine programming still need to be elucidated in order to identify potential target mechanisms for reprogramming strategies [56].

In obese pregnancies, the increase in the levels of proinflammatory cytokines and chemokines and the lipotoxic environment can impact placental oxidative stress and impair mitochondria [57]. A mammal’s placenta requires high oxygen levels from the uterine circulation and massively depends on OXPHOS ATP supply by the mitochondria that need to metabolically comply with gestation stages [53,58,59,60].

In this study, we characterized the placental tissue in relation to pre-pregnancy BMI and the presence of GDM by analyzing mitochondrial function and molecular features, as well as the placental oxidative environment.

The clinical characteristics of the study population were carefully considered. All pregnant women were enrolled at the time of an elective cesarean section in the absence of labor, thus avoiding any bias related to labor-induced molecular and metabolic changes. Both NW and OB groups did not present any associated pathology, except for GDM in OB GDM(+) women. All women were Caucasian, which eluded any genetic contribution to complex disorders [61,62,63]. The study subgroups were homogeneous for all baseline characteristics and neonatal sex, making further comparisons reliable. Possible confounding factors were attentively checked during the statistical analysis and did not affect the molecular variables’ results.

All mothers had received nutritional and lifestyle counseling, particularly on weight gain recommendations during pregnancy, which differed depending on pre-gestational BMI. Indeed, all groups, on average, did not exceed gestational weight gain in accordance with the IOM guidelines, and, therefore, women with obesity gained less weight compared to women with normal weight. In addition to the low-calorie diet recommended for OB GDM(+) women, this allowed them to avoid any pharmacologic therapy (e.g., insulin or metformin). The compliance with IOM guidelines on gestational weight gain might also explain why the newborns of women with obesity, who are frequently heavier, instead had a birth weight similar to those of women with a normal weight [64,65].

Despite that, placental efficiency, estimated by calculating the ratio between neonatal and placental weight, was significantly lower in pregnancies of women with obesity, with an additive effect of GDM, compared to women with a normal weight. Furthermore, placental efficiency was negatively correlated with pre-pregnancy BMI and placental thickness, accounting for a progressive impairment of placental function with the increase in maternal pre-pregnancy BMI. These results are in line with previous data from our group [29,30,31,66,67], confirming a placental insufficiency in obese pregnancies. Additionally, although a hyperdynamic circulation was shown in obese pregnancies, a mild chronic fetoplacental hypoxia was univocally described as a possible consequence of maternal respiratory maladaptation, iron-deficient anemia, and a chronic low-grade inflammatory state resulting in deranged systemic and fetoplacental vascular perfusion [68,69,70].

In line with these data, we detected significantly lower pO_2_ and higher pCO_2_, as well as a trend towards higher lactate concentrations in the umbilical vein of OB vs. NW groups, likely indicating fetal disadvantages and the impairment of developmental programming. This is in line with our previous findings, reporting that fetuses from OB mothers were more hypoxic and acidemic compared to NW mothers [29].

In order to evaluate the placental mitochondrial features, we investigated a range of mitochondrial proteins, as well as placental oxygen consumption and ATP production, accounting for the mitochondrial function. We also evaluated the placental oxidative environment by measuring the protein levels of pro- and anti-oxidant enzymes involved in the cell oxidative balance. We chose SOD2, PON2 (anti-oxidants), and TXNIP (pro-oxidants) as their deregulation can strongly impact mitochondrial function, cell oxidative stress and inflammation, and placental function [71,72,73].

Cyclophilin D (CypD), a peptidyl-prolyl isomerase of the cyclophilin family, is a mitochondrial matrix protein with a critical role in modulating mitochondrial permeability. It is an index of mitochondrial mass. Our data showed no differences in placental CypD protein expression, suggesting no changes in the mitochondrial amount among the placentas of the analyzed groups. However, placental CypD expression was positively correlated to both UV pO_2_ and UV satO_2_ and negatively correlated with UV pCO_2_, accounting for a potential link between the number of placental mitochondria and fetal oxygenation.

Despite no differences in CypD protein expression, the placental oxygen consumption rate was significantly lower in OB GDM(−) compared to NW placentas. Both the coupled OXPHOS respiration and the maximal capacity were impaired, indicating a lower mitochondrial efficiency and an overall reduced bioenergetic spare capacity in obese placentas compared to NW placentas, with a progressive reduction as BMI increased. Accordingly, placental ATP content was significantly lower in both OB GDM(−) and OB GDM(+) compared to NW women and further negatively correlated with pre-pregnancy BMI. This is in accordance with other studies reporting reduced ATP production in pregnancies affected by obesity and GDM, possibly due to the increased oxidative stress, which impairs mitochondrial functions. Sobrevia and colleagues reported that the placentas from GDM women showed higher ROS-induced mtDNA damage and lipid peroxidation, limiting mitochondrial respiration and ATP generation [74]. Other studies described significantly higher ROS generation and peroxidase activity with increasing maternal adiposity, along with lower mitochondrial respiration and deficient ATP generation in the placenta [75,76]. Interestingly, in the present study, placental ATP levels were also positively correlated with placental efficiency, suggesting that ATP deficiency may be linked to placental dysfunction. Moreover, the placental ATP production also correlated with the umbilical vein oxygen saturation, likely accounting for better placental efficiency when ATP availability is increased.

In accordance with a reduced placental ATP production, when comparing the three groups of NW, OB GDM(−), and OB GDM(+), we found a different expression of the mitochondrial complex V, also known as ATP synthase, that couples the dissipation of the proton gradient to the phosphorylation of ADP into ATP. Complex V expression was lower in OB, more so when GDM occurred. Its placental expression was also correlated with umbilical vein glucose, suggesting a role in powering the placental function of nutrient transfer to the fetus.

Taken together, these results suggest that in OB placentas, an altered function and expression of complexes involved in the electron transport system leads to impaired mitochondrial respiration and reduced total placental ATP content. In addition, reduced ATP generation could also likely result from mitochondrial inner membrane cristae disorganization, which we previously reported in OB pregnancies [30].

The protein expression of the pro- and anti-oxidant enzymes that were analyzed in the present study was not different among groups. However, the expression of the anti-oxidant enzyme SOD2 was significantly negatively correlated with maternal pre-pregnancy BMI, suggesting partial impairment of anti-oxidant defenses with increasing BMI, possibly driving a ROS increase in placental cells. Moreover, the pro-oxidant enzyme TXNIP was significantly positively correlated with the placental thickness and negatively correlated with the placental surface, which are morphological characteristics inversely related or directly related, respectively, to the capacity of the placenta to transfer oxygen and nutrients to the fetus. This suggests a relationship between the placental oxidative status and placental efficiency. In accordance with these results, in a previous study, our group showed lower expression of the antioxidant genes *Catalase*, *Glutathione Synthetase,* and *Glutathione Peroxidase 1*, with a significant relation between their expression and placental efficiency, thus reinforcing the hypothesis of an alteration of anti-oxidant defenses in relation to pre-pregnancy maternal BMI and to placental inefficiency. This is also in agreement with previously reported changes in the placental metabolic profile in obese pregnancies, showing differences in metabolites involved in antioxidant defenses and energy production [29,30].

We did not find any difference in molecular data depending on neonatal sex. However, the small size of the population, when divided into females and males, could be the cause of a failure to detect differences between the two neonatal sexes. The limited sample size may represent a limitation of this study. Nevertheless, the study population was carefully selected, thus excluding several pregnancies, in order to minimize the confounding effect of parameters that could have influenced the results, such as labor or the use of therapies. This led to the selection of a lower number of patients, who nevertheless showed substantial differences in numerous study parameters. Another possible limitation of this study is the lack of data on possible maternal supplement use, which could have impacted the intrauterine environment. However, all women underwent the same antenatal nutritional counseling, which is routine in the obstetric clinic where the women were enrolled, thus exposing all women to the same potential biases. Finally, although interesting and significant, correlations should be taken with caution, as they do not demonstrate causality. Nevertheless, they are indicative of an association providing clues for the interpretation of the data and representing an initial pointer for further investigation.

## 5. Conclusions

Pregnancy is a period of great sensitivity for the developing individual, and the maternal nutritional status can strongly influence fetal programming and the short and long-term health of the unborn child [77,78]. In the present study, the simultaneous analysis of multiple factors relating to the bioenergetic and oxidative state of the placenta allowed us to paint a multifaceted picture of placental characteristics in pregnancies of women with different BMI or affected by gestational diabetes. Overall, the results of this study suggest alterations of placental bioenergetics in pregnancies of women with obesity, which is linked with defects of the pro/anti-oxidative machinery, possibly leading to placental dysfunction and to the impairment of oxygen and nutrient transport to the fetus. An unbalanced pregestational nutritional status can, therefore, compromise placental mitochondrial functions, impacting placental efficiency and, consequently, maternal–fetal exchanges. To our knowledge, this is the first study simultaneously analyzing placental mitochondrial proteins, functions, and products, as well as the pro/anti-oxidative machinery, in relation to pregestational BMI and to the placental efficiency of pregnant women with or without GDM. These results fill a further gap in the knowledge of placental dysfunction in the presence of maternal obesity, paving the way for the identification of biomarkers and therapeutic agents or targets for the diagnosis and treatment of placental dysfunction. Further studies will be necessary to unravel specific molecular processes below this observation, also taking into account the possible contribution of other soluble factors in the obese environment, such as the increase in the circulating and placental levels of proinflammatory cytokines and chemokines.

## Figures and Tables

**Table 1:** Clinical characteristics of the study population: 17 NW, 19 OB GDM(−), 10 OB GDM(+). Data are presented as average ± standard deviation. BMI: Body Mass Index; GWG: Gestational Weight Gain; Placental efficiency N/P: neonatal weight/placental weight ratio; n.s.: not significant. * *p* < 0.05, ** *p* < 0.01, *** *p* < 0.001 vs. NW.

		Average ± Standard Deviation	*p*
pregestational BMI (kg/m^2^)	NW	21.64 ± 1.93	**<0.001**
	OB_GDM(−)	33.48 ± 2.69 ***	
	OB_GDM(+)	34.38 ± 2.34 ***	
	*OB_all*	33.80 ± 2.57 ***	**<0.001**
maternal age (years)	NW	34.3 ± 3.6	n.s.
	OB_GDM(−)	30.2 ± 6.0	
	OB_GDM(+)	34.8 ± 4.4	
	*OB_all*	31.8 ± 5.9	n.s.
GWG (kg)	NW	12.19 ± 2.64	**0.017**
	OB_GDM(−)	8.39 ± 5.36 *	
	OB_GDM(+)	6.17 ± 6.56 **	
	*OB_all*	7.65 ± 5.76 **	**0.006**
nulliparous, n (%)	NW	4 (23%)	n.s.
	OB_GDM(−)	1 (5%)	
	OB_GDM(+)	2 (20%)	
	*OB_all*	3 (10%)	n.s.
gestational age (weeks)	NW	39.1 ± 0.2	n.s.
	OB_GDM(−)	39.1 ± 0.3	
	OB_GDM(+)	39.1 ± 0.2	
	*OB_all*	39.1 ± 0.3	n.s.
neonatal weight, N (g)	NW	3468 ± 285	n.s.
	OB_GDM(−)	3374 ± 332	
	OB_GDM(+)	3329 ± 346	
	*OB_all*	3358 ± 331	n.s.
neonatal sex, females/males (n)	NW	9/8	n.s.
	OB_GDM(−)	6/12	
	OB_GDM(+)	5/5	
	*OB_all*	11/17	n.s.
placental major diameter (cm)	NW	18.8 ± 2.0	n.s.
	OB_GDM(−)	18.4 ± 1.6	
	OB_GDM(+)	19.9 ± 3.7	
	*OB_all*	18.9 ± 2.5	n.s.
placental minor diameter (cm)	NW	15.7 ± 1.9	n.s.
	OB_GDM(−)	15.4 ± 2.2	
	OB_GDM(+)	15.7 ± 1.0	
	*OB_all*	15.5 ± 1.9	n.s.
placental weight, P (g)	NW	446.4 ± 76.4	n.s.
	OB_GDM(−)	488.2 ± 76.28	
	OB_GDM(+)	509.0 ± 65.9	
	*OB_all*	495.6 ± 72.2 *	**0.04**
placental surface (cm^3^)	NW	234.7 ± 50.0	n.s.
	OB_GDM(−)	224.0 ± 44.5	
	OB_GDM(+)	245.6 ± 49.6	
	*OB_all*	231.2 ± 46.5	n.s.
placental thickness (cm)	NW	1.97 ± 0.46	n.s.
	OB_GDM(−)	2.24 ± 0.46	
	OB_GDM(+)	2.16 ± 0.54	
	*OB_all*	2.21 ± 0.48	n.s.
placental efficiency N/P	NW	7.99 ± 1.38	**0.002**
	OB_GDM(−)	7.03 ± 1.04 *	
	OB_GDM(+)	6.25 ± 0.63 ***	
	*OB_all*	6.77 ± 0.98 *	**0.04**

**Table 2:** Maternal and fetal blood values in the study population: 17 NW, 19 OB GDM(−), 10 OB GDM(+). Data are presented as average ± standard deviation. UA: Umbilical Arterial blood; UV: Umbilical Vein blood; satO_2_: oxygen saturation; OGTT: Oral Glucose Tolerance Test; n.s.: not significant. * *p* < 0.05 vs. NW; †† *p* < 0.01; ††† *p* < 0.001 vs. OB GDM(+).

		Average ± Standard Deviation	*p*
pH UA	NW	7.32 ± 0.049	n.s.
	OB_GDM(−)	7.29 ± 0.052	
	OB_GDM(+)	7.31 ± 0.049	
	*OB_all*	7.30 ± 0.052	n.s.
pH UV	NW	7.37 ± 0.044	n.s.
	OB_GDM(−)	7.34 ± 0.060	
	OB_GDM(+)	7.35 ± 0.044	
	*OB_all*	7.34 ± 0.055	n.s.
pO_2_ UV (mmHg)	NW	26.44 ± 5.08	n.s.
	OB_GDM(−)	21.33 ± 8.72	
	OB_GDM(+)	21.38 ± 6.39	
	*OB_all*	21.35 ± 7.69 *	**0.045**
satO_2_ UV (g/dL)	NW	54.57 ± 16.74	n.s.
	OB_GDM(−)	39.98 ± 23.69	
	OB_GDM(+)	41.57 ± 18.98	
	*OB_all*	40.54 ± 21.54	n.s.
pCO_2_ UV (mmHg)	NW	41.90 ± 5.64	n.s.
	OB_GDM(−)	46.85 ± 8.66	
	OB_GDM(+)	47.17 ± 6.09	
	*OB_all*	46.97 ± 7.63 *	**0.047**
glucose UV (mg/dL)	NW	66.62 ± 7.46	n.s.
	OB_GDM(−)	62.83 ± 9.35	
	OB_GDM(+)	64.25 ± 9.38	
	*OB_all*	63.40 ± 9.14	n.s.
lactate UV (mmol/L)	NW	1.59 ± 0.37	n.s.
	OB_GDM(−)	1.90 ± 0.70	
	OB_GDM(+)	1.80 ± 0.45	
	*OB_all*	1.86 ± 0.60	n.s.
OGTT I (mg/dL)	NW	83.09 ± 3.51	n.s.
	OB_GDM(−)	81.00 ± 7.20	
	OB_GDM(+)	84.63 ± 13.23	
	*OB_all*	82.53 ± 10.01	n.s.
OGTT II (mg/dL)	NW	130.55 ± 21.68	**0.015**
	OB_GDM(−)	117.64 ± 30.77 ††	
	OB_GDM(+)	159.00 ± 33.91	
	*OB_all*	135.05 ± 37.60	n.s.
OGTT III (mg/dL)	NW	108.82 ± 32.74 ††	**0.003**
	OB_GDM(−)	99.82 ± 24.26 †††	
	OB_GDM(+)	159.63 ± 32.89	
	*OB_all*	125.00 ± 40.84	n.s.

## Data Availability

The original data presented in the study are included in the article/Supplementary Material; further inquiries can be directed to the corresponding authors.

## Supplementary Materials

The following supporting information can be downloaded at https://www.mdpi.com/article/10.3390/antiox13070858/s1, Table S1: Primary antibodies details; and Table S2: Detailed protein expression data.

## References

[1] World Obesity Federation World Obesity Atlas 2023. https://www.worldobesity.org/resources/resource-library/world-obesity-atlas-2023.

[2] Creanga A.A., Catalano P.M., Bateman B.T. (2022). Obesity in pregnancy. N. Engl. J. Med..

[3] Hanson M.A., Bardsley A., De-Regil L.M., Moore S.E., Oken E., Poston L., Ma R.C., McAuliffe F.M., Maleta K., Purandare C.N. (2015). The International Federation of Gynecology and Obstetrics (FIGO) recommendations on adolescent, preconception, and maternal nutrition: “Think Nutrition First”. Int. J. Gynaecol. Obstet..

[4] Thiele K., Diao L., Arck P.C. (2018). Immunometabolism, pregnancy, and nutrition. Semin. Immunopathol..

[5] Goyal D., Limesand S.W., Goyal R. (2019). Epigenetic responses and the developmental origins of health and disease. J. Endocrinol..

[6] Zhang C.X.W., Candia A.A., Sferruzzi-Perri A.N. (2024). Placental inflammation, oxidative stress, and fetal outcomes in maternal obesity. Trends Endocrinol. Metab..

[7] O’Reilly J.R., Reynolds R.M. (2013). The risk of maternal obesity to the long-term health of the offspring. Clin. Endocrinol..

[8] Hutchins F., Krafty R., El Khoudary S.R., Catov J., Colvin A., Barinas-Mitchell E., Brooks M.M. (2021). Gestational weight gain and long-term maternal obesity risk: A multiple-bias analysis. Epidemiology.

[9] Gohir W., Ratcliffe E.M., Sloboda D.M. (2015). Of the bugs that shape us: Maternal obesity, the gut microbiome, and long-term disease risk. Pediatr. Res..

[10] Barker D.J., Winter P.D., Osmond C., Margetts B., Simmonds S.J. (1989). Weight in infancy and death from ischaemic heart disease. Lancet.

[11] Agosti M., Tandoi F., Morlacchi L., Bossi A. (2017). Nutritional and metabolic programming during the first thousand days of life. Pediatr. Med. Chir..

[12] Zavatta A., Parisi F., Mandò C., Scaccabarozzi C., Savasi V.M., Cetin I. (2023). Role of inflammaging on the reproductive function and pregnancy. Clin. Rev. Allergy Immunol..

[13] Kerr B., Leiva A., Farías M., Contreras-Duarte S., Toledo F., Stolzenbach F., Silva L., Sobrevia L. (2018). Foetoplacental epigenetic changes associated with maternal metabolic dysfunction. Placenta.

[14] Sugino K.Y., Janssen R.C., McMahan R.H., Zimmerman C., Friedman J.E., Jonscher K.R. (2024). Vertical transfer of maternal gut microbes to offspring of western diet-fed dams drives reduced levels of tryptophan metabolites and postnatal innate immune response. Nutrients.

[15] Martin S.L., Zhang L., Callahan M.L., Bahorski J., Lewis C.E., Hidalgo B.A., Durant N., Harper L.M., Battarbee A.N., Habegger K. (2022). Mother-child cardiometabolic health 4–10 years after pregnancy complicated by obesity with and without gestational diabetes. Obes. Sci. Pract..

[16] Serati A., Novielli C., Anelli G.M., Mandalari M., Parisi F., Cetin I., Paleari R., Mandò C. (2023). Characterization of maternal circulating microRNAs in obese pregnancies and gestational diabetes mellitus. Antioxidants.

[17] Quotah O.F., Poston L., Flynn A.C., White S.L. (2022). Metabolic profiling of pregnant women with obesity: An exploratory study in women at greater risk of gestational diabetes. Metabolites.

[18] Catalano P.M. (2010). The impact of gestational diabetes and maternal obesity on the mother and her offspring. J. Dev. Orig. Health Dis..

[19] Musumeci A., McElwain C.J., Manna S., McCarthy F., McCarthy C. (2024). Exposure to gestational diabetes mellitus increases subclinical inflammation mediated in part by obesity. Clin. Exp. Immunol..

[20] Rees A., Richards O., Allen-Kormylo A., Jones N., Thornton C.A. (2022). Maternal body mass index is associated with an altered immunological profile at 28 weeks of gestation. Clin. Exp. Immunol..

[21] Tossetta G., Fantone S., Gesuita R., Di Renzo G.C., Meyyazhagan A., Tersigni C., Scambia G., Di Simone N., Marzioni D. (2022). HtrA1 in gestational diabetes mellitus: A possible biomarker?. Diagnostics.

[22] Luo R., Fell D.B., Corsi D.J., Taljaard M., Wen S.W., Walker M.C. (2024). Temporal trends in gestational diabetes mellitus and associated risk factors in Ontario, Canada, 2012–2020: A population-based cohort study. J. Obstet. Gynaecol. Can..

[23] Mackeen A.D., Boyd V.E., Schuster M., Young A.J., Gray C., Angras K. (2024). The impact of prepregnancy body mass index on pregnancy and neonatal outcomes. J. Osteopath. Med..

[24] Lappas M., Yee K., Permezel M., Rice G.E. (2005). Release and regulation of leptin, resistin and adiponectin from human placenta, fetal membranes, and maternal adipose tissue and skeletal muscle from normal and gestational diabetes mellitus-complicated pregnancies. J. Endocrinol..

[25] Perugini J., Di Mercurio E., Tossetta G., Severi I., Monaco F., Reguzzoni M., Tomasetti M., Dani C., Cinti S., Giordano A. (2019). Biological effects of ciliary neurotrophic factor on hMADS adipocytes. Front. Endocrinol..

[26] Álvarez D., Muñoz Y., Ortiz M., Maliqueo M., Chouinard-Watkins R., Valenzuela R. (2020). Impact of maternal obesity on the metabolism and bioavailability of polyunsaturated fatty acids during pregnancy and breastfeeding. Nutrients.

[27] Castro-Rodríguez D.C., Rodríguez-González G.L., Menjivar M., Zambrano E. (2020). Maternal interventions to prevent adverse fetal programming outcomes due to maternal malnutrition: Evidence in animal models. Placenta.

[28] Hebert J.F., Myatt L. (2021). Placental mitochondrial dysfunction with metabolic diseases: Therapeutic approaches. Biochim. Biophys. Acta Mol. Basis Dis..

[29] Bianchi C., Taricco E., Cardellicchio M., Mandò C., Massari M., Savasi V., Cetin I. (2021). The role of obesity and gestational diabetes on placental size and fetal oxygenation. Placenta.

[30] Diceglie C., Anelli G.M., Martelli C., Serati A., Lo Dico A., Lisso F., Parisi F., Novielli C., Paleari R., Cetin I. (2021). Placental antioxidant defenses and autophagy-related genes in maternal obesity and gestational diabetes mellitus. Nutrients.

[31] Anelli G.M., Cardellicchio M., Novielli C., Antonazzo P., Mazzocco M.I., Cetin I., Mandò C. (2018). Mitochondrial content and hepcidin are increased in obese pregnant mothers. J. Matern. Fetal Neonatal Med..

[32] Myatt L., Maloyan A. (2016). Obesity and placental function. Semin. Reprod. Med..

[33] Howell K.R., Powell T.L. (2017). Effects of maternal obesity on placental function and fetal development. Reproduction.

[34] Duffley E., Grynspan D., Scott H., Lafrenière A., Borba Vieira de Andrade C., Bloise E., Connor K.L. (2024). Gestational age, infection, and suboptimal maternal prepregnancy BMI independently associate with placental histopathology in a cohort of pregnancies without major maternal comorbidities. J. Clin. Med..

[35] Hietalati S., Pham D., Arora H., Mochizuki M., Santiago G., Vaught J., Lin E.T., Mestan K.K., Parast M., Jacobs M.B. (2024). Placental pathology and fetal growth outcomes in pregnancies complicated by maternal obesity. Int. J. Obes..

[36] Holland O., Dekker Nitert M., Gallo L.A., Vejzovic M., Fisher J.J., Perkins A.V. (2017). Review: Placental mitochondrial function and structure in gestational disorders. Placenta.

[37] Lu M., Sferruzzi-Perri A.N. (2021). Placental mitochondrial function in response to gestational exposures. Placenta.

[38] Fisher J.J., Bartho L.A., Perkins A.V., Holland O.J. (2020). Placental mitochondria and reactive oxygen species in the physiology and pathophysiology of pregnancy. Clin. Exp. Pharmacol. Physiol..

[39] Wang A., Li Z., Zhang D., Chen C., Zhang H. (2024). Excessive ER-phagy mediated by FAM134B contributes to trophoblast cell mitochondrial dysfunction in preeclampsia. Acta Biochim. Biophys. Sin..

[40] Barrientos G., Schuman M.L., Landa M.S., Robello E., Incardona C., Conrad M.L., Galleano M., García S.I. (2024). Therapeutic effect of alpha lipoic acid in a rat preclinical model of preeclampsia: Focus on maternal signs, fetal growth and placental function. Antioxidants.

[41] Bonora M., Patergnani S., Rimessi A., De Marchi E., Suski J.M., Bononi A., Giorgi C., Marchi S., Missiroli S., Poletti F. (2012). ATP synthesis and storage. Purinergic Signal..

[42] Pan M., Zhou J., Wang J., Cao W., Li L., Wang L. (2023). The role of placental aging in adverse pregnancy outcomes: A mitochondrial perspective. Life Sci..

[43] Hadlock F.P., Shah Y.P., Kanon D.J., Lindsey J.V. (1992). Fetal crown-rump length: Reevaluation of relation to menstrual age (5–18 weeks) with high-resolution real-time US. Radiology.

[44] Rasmussen K.M., Yaktine A.L. (2009). Institute of Medicine (US) and National Research Council (US) committee to reexamine IOM pregnancy weight guidelines. Weight Gain during Pregnancy: Reexamining the Guidelines.

[45] Hod M., Kapur A., Sacks D.A., Hadar E., Agarwal M., Di Renzo G.C., Cabero Roura L., McIntyre H.D., Morris J.L., Divakar H. (2015). The International Federation of Gynecology and Obstetrics (FIGO) initiative on gestational diabetes mellitus: A pragmatic guide for diagnosis, management, and care. Int. J. Gynaecol. Obstet..

[46] International Association of Diabetes and Pregnancy Study Groups Consensus Panel (2010). International association of diabetes and pregnancy study groups recommendations on the diagnosis and classification of hyperglycemia in pregnancy. Diabetes Care.

[47] Casati D., Lanna M., Mando C., Zavatta A., Nelva Stellio L., Faiola S., Laoreti A., Anelli G.M., Cetin I. (2023). Fetal oxygen and glucose utilization of uncomplicated monochorionic twins: Adapting to the intrauterine environment. Placenta.

[48] Anelli G.M., Mandò C., Letizia T., Mazzocco M.I., Novielli C., Lisso F., Personeni C., Vago T., Cetin I. (2019). Placental ESRRG-CYP19A1 expressions and circulating 17-beta estradiol in IUGR pregnancies. Front. Pediatr..

[49] Giovarelli M., Serati A., Zecchini S., Guelfi F., Clementi E., Mandò C. (2023). Cryopreserved placental biopsies maintain mitochondrial activity for high-resolution respirometry. Mol. Med..

[50] Chida J., Yamane K., Takei T., Kido H. (2012). An efficient extraction method for quantitation of adenosine triphosphate in mammalian tissues and cells. Anal. Chim. Acta..

[51] Pedrotti S., Caccia R., Neguembor M.V., Garcia-Manteiga J.M., Ferri G., de Palma C., Canu T., Giovarelli M., Marra P., Fiocchi A. (2019). The Suv420h histone methyltransferases regulate PPAR-gamma and energy expenditure in response to environmental stimuli. Sci. Adv..

[52] Carli S., Chaabane L., Butti C., De Palma C., Aimar P., Salio C., Vignoli A., Giustetto M., Landsberger N., Frasca A. (2021). In vivo magnetic resonance spectroscopy in the brain of Cdkl5 null mice reveals a metabolic profile indicative of mitochondrial dysfunctions. J. Neurochem..

[53] Holland O.J., Hickey A.J.R., Alvsaker A., Moran S., Hedges C., Chamley L.W., Perkins A.V. (2017). Changes in mitochondrial respiration in the human placenta over gestation. Placenta.

[54] Sormunen-Harju H., Huvinen E., Girchenko P.V., Kajantie E., Villa P.M., Hämäläinen E.K., Lahti-Pulkkinen M., Laivuori H., Räikkönen K., Koivusalo S.B. (2023). Metabolomic profiles of nonobese and obese women with gestational diabetes. J. Clin. Endocrinol. Metab..

[55] Musa E., Salazar-Petres E., Arowolo A., Levitt N., Matjila M., Sferruzzi-Perri A.N. (2023). Obesity and gestational diabetes independently and collectively induce specific effects on placental structure, inflammation and endocrine function in a cohort of South African women. J. Physiol..

[56] Hsu C.N., Tain Y.L. (2019). The Good, the Bad, and the Ugly of pregnancy nutrients and developmental programming of adult disease. Nutrients.

[57] Kelly A.C., Powell T.L., Jansson T. (2020). Placental function in maternal obesity. Clin. Sci..

[58] Schneider H. (2000). Placental oxygen consumption. Part II: In vitro studies—A review. Placenta.

[59] Burton G.J. (2009). Oxygen, the Janus gas; its effects on human placental development and function. J. Anat..

[60] Hung T.H., Skepper J.N., Charnock-Jones D.S., Burton G.J. (2002). Hypoxia-reoxygenation: A potent inducer of apoptotic changes in the human placenta and possible etiological factor in preeclampsia. Circ. Res..

[61] Kenney M.C., Chwa M., Atilano S.R., Falatoonzadeh P., Ramirez C., Malik D., Tarek M., Del Carpio J.C., Nesburn A.B., Boyer D.S. (2014). Molecular and bioenergetic differences between cells with African versus European inherited mitochondrial DNA haplogroups: Implications for population susceptibility to diseases. Biochim. Biophys. Acta (BBA) Mol. Basis Dis..

[62] Gómez-Durán A., Pacheu-Grau D., López-Gallardo E., Díez-Sánchez C., Montoya J., López-Pérez M.J., Ruiz-Pesini E. (2010). Unmasking the causes of multifactorial disorders: OXPHOS differences between mitochondrial haplogroups. Hum. Mol. Genet..

[63] Neikirk K., Kabugi K., Mungai M., Kula B., Smith N., Hinton A.O. (2024). Ethnicity-related differences in mitochondrial regulation by insulin stimulation in diabetes. J. Cell Physiol..

[64] Dalfra M.G., Burlina S., Lapolla A. (2022). Weight gain during pregnancy: A narrative review on the recent evidences. Diabetes Res. Clin. Pract..

[65] Pauwels S., Ghosh M., Duca R.C., Bekaert B., Freson K., Huybrechts I., Langie S.A.S., Koppen G., Devlieger R., Godderis L. (2017). Maternal intake of methyl-group donors affects DNA methylation of metabolic genes in infants. Clin. Epigenetics.

[66] Mandò C., Abati S., Anelli G.M., Favero C., Serati A., Dioni L., Zambon M., Albetti B., Bollati V., Cetin I. (2022). Epigenetic profiling in the saliva of obese pregnant women. Nutrients.

[67] Assi E., D’Addio F., Mandò C., Maestroni A., Loretelli C., Ben Nasr M., Usuelli V., Abdelsalam A., Seelam A.J., Pastore I. (2020). Placental proteome abnormalities in women with gestational diabetes and large-for-gestational-age newborns. BMJ Open Diabetes Res. Care..

[68] Patel D., Avesani M., Johnson M.R., Di Salvo G., Savvidou M.D. (2024). Maternal cardiovascular adaptation to pregnancy in obese pregnant women. Acta Obstet. Gynecol. Scand..

[69] Åmark H., Sirotkina M., Westgren M., Papadogiannakis N., Persson M. (2020). Is obesity in pregnancy associated with signs of chronic fetal hypoxia?. Acta Obstet. Gynecol. Scand..

[70] Pardo F., Subiabre M., Fuentes G., Toledo F., Silva L., Villalobos-Labra R., Sobrevia L. (2019). Altered foetoplacental vascular endothelial signalling to insulin in diabesity. Mol. Aspects Med..

[71] Silveira M.A.D., Marcondes J.P.C., Lara J.R., Scarano W.R., Calderón I.M.P., Rudge M.V.C., Salvadori D.M.F. (2019). Mitochondrial-related gene associated to obesity can be modulated by in utero hyperglycemic environment. Reprod. Toxicol..

[72] Alwarfaly S., Abdulsid A., Hanretty K., Lyall F. (2014). Paraoxonase 2 protein is spatially expressed in the human placenta and selectively reduced in labour. PLoS ONE.

[73] Sa R., Ma J., Yang J., Li D.F., Du J., Jia J.C., Li Z.Y., Huang N., A L., Sha R. (2023). High TXNIP expression accelerates the migration and invasion of the GDM placenta trophoblast. BMC Pregnancy Childbirth.

[74] Sobrevia L., Valero P., Grismaldo A., Villalobos-Labra R., Pardo F., Subiabre M., Armstrong G., Toledo F., Vega S., Cornejo M. (2020). Mitochondrial dysfunction in the fetoplacental unit in gestational diabetes mellitus. Biochim. Biophys. Acta Mol. Basis Dis..

[75] Rodríguez-Cano A.M., Calzada-Mendoza C.C., Estrada-Gutierrez G., Mendoza-Ortega J.A., Perichart-Perera O. (2020). Nutrients, mitochondrial function, and perinatal health. Nutrients.

[76] Mele J., Muralimanoharan S., Maloyan A., Myatt L. (2014). Impaired mitochondrial function in human placenta with increased maternal adiposity. Am. J. Physiol. Endocrinol. Metabol..

[77] Massari M., Novielli C., Mandò C., Di Francesco S., Della Porta M., Cazzola R., Panteghini M., Savasi V., Maggini S., Schaefer E. (2020). Multiple micronutrients and docosahexaenoic acid supplementation during pregnancy: A randomized controlled study. Nutrients.

[78] Tiffon C. (2018). The impact of nutrition and environmental epigenetics on human health and disease. Int. J. Mol. Sci..

