# Supplementary material for: Placental Bioenergetics and Antioxidant Homeostasis in Maternal Obesity and Gestational Diabetes
Source: Antioxidants (Basel). 2024 Jul 18;13(7):858. doi: 10.3390/antiox13070858 (PMC11273840; doi:10.3390/antiox13070858)
Supplement: Supplementary file 1 [file antioxidants-13-00858-s001.zip › antioxidants-3081353-supplementary.pdf]

Supplementary

Table S1. Primary antibodies details

target	ref n.	company	dilution
Paraoxonase 2 (PON2)	PA5-25663	Thermo Fisher Scientific (Waltham, MA, USA)	1:1000
Superoxide Dismutase 2 (SOD2)	611580	BD Transduction Laboratories (Milano, Italy)	1:2000
Thioredoxin Interacting Protein (TXNIP)	40-3700	Invitrogen, Thermo Fisher Scientific	1:250
Cyclophilin D (CypD)	45-5900	Thermo Fisher Scientific	1:1000
OxPhos	45-8099	Thermo Fisher Scientific	1:1000
Actin	sc-1616-r	Santa Cruz Biotechnology (Dallas, TX, USA)	1:200

Table S2. Detailed placental protein expression data. Data are presented as average ± standard deviation. ** $p < 0.01$ vs NW

		average ± standard deviation	*p*
Cyclophilin D	NW	1.75 ± 2.17	n.s.
	OB_GDM(-)	1.89 ± 2.03	
	OB_GDM(+)	1.32± 0.44	
	OB_all	*1.70 ± 1.68*	*n.s.*
Mitochondrial Complex I	NW	4.38 ± 3.21	n.s.
	OB_GDM(-)	4.95 ± 6.54	
	OB_GDM(+)	5.07 ± 5.92	
	OB_all	*5.00 ± 6.19*	*n.s.*
Mitochondrial Complex II	NW	7.61 ± 10.74	n.s.
	OB_GDM(-)	9.21 ± 13.93	
	OB_GDM(+)	16.63 ± 29.32	
	OB_all	*11.96 ± 20.74*	*n.s.*
Mitochondrial Complex III	NW	10.21 ± 19.59	n.s.
	OB_GDM(-)	8.30 ± 13.07	
	OB_GDM(+)	14.60 ± 28.08	
	OB_all	10.64 ± 19.69	*n.s.*
Mitochondrial Complex IV	NW	10.45 ± 17.00	n.s.
	OB_GDM(-)	10.83 ± 18.31	
	OB_GDM(+)	17.97 ± 31.29	
	OB_all	*13.47 ± 23.61*	*n.s.*
Mitochondrial Complex V	NW	4.71 ± 2.86	**p=0.05**
	OB_GDM(-)	3.88 ± 2.11	
	OB_GDM(+)	2.91 ± 2.68**	
	OB_all	*3.56 ± 2.31*	*n.s.*
SOD2	NW	10.32 ± 9.85	n.s.
	OB_GDM(-)	7.24 ± 5.16	
	OB_GDM(+)	5.18 ± 4.15	
	OB_all	*6.55 ± 4.87*	*n.s.*
PON2	NW	15.17 ± 6.75	n.s.
	OB_GDM(-)	17.91 ± 13.04	
	OB_GDM(+)	17.33 ± 8.89	
	OB_all	*17.71 ± 11.65*	*n.s.*
TXNIP	NW	13.87 ± 12.27	

	OB_GDM(-)	20.82 ± 18.38	n.s.
	OB_GDM(+)	15.24 ± 17.08	
	OB_all	*18.96 ± 17.83*	*n.s.*
